# Risk factors and socioeconomic determinants of falls among older adults

**DOI:** 10.3389/fpubh.2025.1571312

**Published:** 2025-03-13

**Authors:** Agnieszka Maruszewska, Tadeusz Ambroży, Łukasz Rydzik

**Affiliations:** ^1^Department of Physiotherapy, Faculty of Health Sciences, Vincent Pol University, Lublin, Poland; ^2^Department of Sport Theory and Motor Skills, Institute of Sport Sciences, University of Physical Culture in Kraków, Kraków, Poland

**Keywords:** falls, older adults, major geriatric syndrome, socioeconomic factors, risk assessment

## Abstract

**Background:**

Falls are included in this category due to their high prevalence among people over 60 years of age. The aim of this study is to analyze the relationship between the frequency of falls and selected socioeconomic factors.

**Methods:**

The study was conducted among 351 patients of a rehabilitation clinic and center. The research method was a structured diagnostic survey using the Falls Risk Assessment Tool (FRAT) to collect data on fall incidence and associated risk factors.

**Results:**

A significant relationship was found between the age of the participants, their level of education (*p* = 0.00015), and the type of work previously performed (*p* = 0.00039) and the frequency of falls. The frequency of falls increased with age, and falls were more common among less educated individuals and those who previously performed physical work. A significant relationship was also found between marital status (*p* = 0.00039), material status (*p* = 0.004), and the number of people in the household (*p* = 0.002) and the frequency of falls. Falls were most frequent among widowed individuals and those with poorer financial situations. People living alone and those living in households with more than two members experienced falls more often.

**Conclusions:**

These findings suggest that educational background and financial constraints may contribute to fall risk, emphasizing the need for targeted fall prevention programs among vulnerable populations.

## 1 Introduction

In older people, the presence of major geriatric problems is often observed. These include, among others, urinary and fecal incontinence, vision and hearing impairments, dementia syndromes, depression, iatrogenic syndromes, frailty syndrome, and balance disorders leading to falls. The presence of these multifactorial disorders in seniors results in the loss of autonomy and control over life and/or poses significant psychological, physical, organizational, and economic burdens on the family or care system (1). Falls are included in this category due to their high prevalence among individuals over the age of 60. In this study, a fall was defined as “an event resulting in an unintentional change in position to a lower level, excluding intentional movements such as sitting down.” Participants were explicitly informed about this definition before completing the questionnaire. This threshold was chosen based on epidemiological studies indicating a sharp increase in fall risk beginning at age 60 (2).

Epidemiological studies indicate that falls and their consequences are the leading causes of injuries, hospitalizations, and even deaths among older adults (3, 4). Beyond medical consequences, falls generate immense social and personal costs, involving pain, loss of confidence, significant reduction in quality of life, or disability (5). Falls may also serve as a critical signal indicating the presence of other severe illnesses, whose symptoms in this age group are often less pronounced than in younger patients. Chronic diseases were classified as severe if they significantly impaired mobility or required ongoing medical treatment, based on the WHO classification of chronic illnesses (6). Examples include urinary tract infections or pneumonia manifesting as falls (7). The vast majority of falls are multifactorially determined (8). Falls are perceived as a general impairment of functioning and are considered a taboo topic, with the affected individuals often reluctant to admit to them or attempting to downplay the severity of the problem (9). Socioeconomic factors play a crucial role in the risk of falls among older adults, as financial constraints, educational background, and access to healthcare services influence their ability to implement fall prevention strategies. Older adults living in domestic environments do not always report falls, and healthcare workers do not always inquire about them unless visible consequences are present. Seniors typically mention a fall only if it results in significant consequences such as fractures or injuries (10, 11). However, in all individuals aged 65 and older, asking about falls should be a routine part of medical history-taking (12). Reducing the risk of falls in older people is one of the most critical challenges in contemporary geriatrics (13). Falls are not only a leading cause of morbidity among older adults but also a significant barrier to successful rehabilitation. In geriatric rehabilitation, the prevention and management of falls are crucial to restoring mobility, independence, and quality of life. Falls often lead to fractures, prolonged hospital stays, and increased reliance on caregivers, further complicating the rehabilitation process. Understanding the factors contributing to falls can inform targeted interventions in rehabilitation settings, ensuring a more holistic approach to older care. It is estimated that among those aged 65 and older, at least one fall per year occurs in 50.0%−67.0% of residents in care homes, 33.0% of individuals living independently, and 20.0% of hospitalized patients (4, 14–16). Nearly half of those who experience a fall will fall again within a year (17). The incidence of falls in the older population gradually increases with age (18, 19). While most falls do not result in bodily harm, over 30.0% lead to injuries requiring medical assistance or limiting activity for at least 1 day, and 10.0%−15.0% of falls among individuals living in their own homes result in severe injuries (20, 21). Despite their high prevalence, falls remain one of the least studied and monitored public health issues in Poland. This challenge primarily concerns public health specialists and institutions (22). There is a lack of current research assessing the occurrence of falls in Poland. Although international studies have examined the relationship between falls and various factors, the specific characteristics of the Polish healthcare system, social structure, and socioeconomic disparities may influence fall risks uniquely. Therefore, localized studies are essential to provide actionable data tailored to the Polish population. The present study addresses this knowledge gap, aiming to evaluate the frequency of falls among older adults. The focus on the frequency of falls rather than their occurrence was dictated by the limited availability of data on recurrent falls in Poland and their relationship with socioeconomic factors. Previous studies have often addressed other determinants, such as health status or physical activity, leaving socioeconomic aspects underexplored.

The objective of the study was to assess the frequency of falls among individuals over 60 years of age and analyze the relationships between the frequency of falls and selected socioeconomic factors such as age, gender, place of residence, level of education, marital status, primary source of income, number of household members, and type of work per-formed.

## 2 Materials and methods

The study was conducted in accordance with the Declaration of Helsinki, with approval obtained from the Bioethics Committee of the Institute of Rural Health named after W. Chodzki in Lublin (Resolution No. 22/A/2019). All participants were informed about the principles and purpose of the study and provided written consent to participate. To minimize recall bias, participants were asked to report falls within the last 12 months, a time frame commonly used in epidemiological studies. Additionally, data collection was conducted through structured interviews to improve accuracy and consistency.

### 2.1 Participants

**Figure 1:**
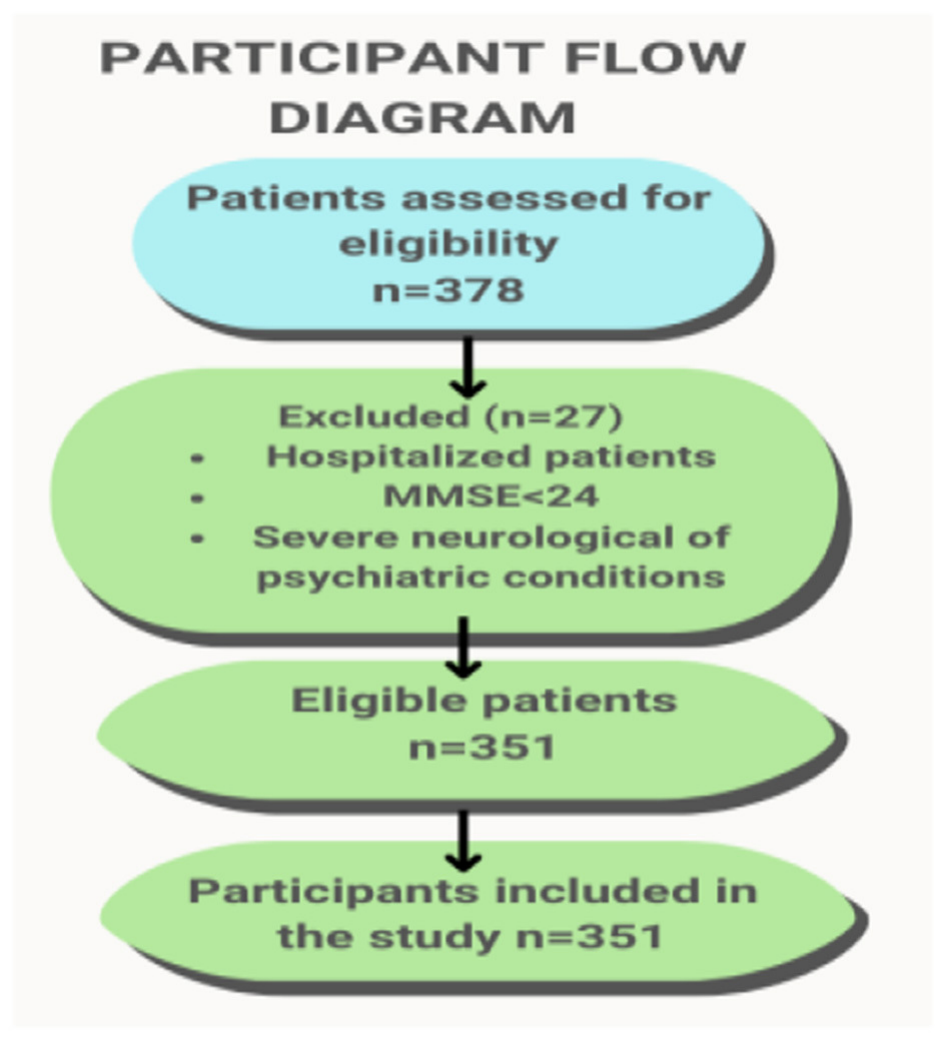
Participant flow diagram illustrating.

The study began with a group of 378 patients, of whom 351 were included in the analysis after applying the exclusion criteria. The required sample size was determined based on an estimated fall incidence of ~30% in older adults, a confidence level of 95%, and a margin of error of 5%. A convenience sampling technique was used to recruit participants from rehabilitation centers. This final group consisted of 351 patients (76.9% women and 23.1% men) recruited from the Rehabilitation Clinic with the Neuro-logical Rehabilitation Subdivision and the Rehabilitation Center of the Institute of Rural Health (Figure 1).

The Table 1 below summarizes the characteristics of the study group, including age, gender, place of residence, education level, type of work, and the number of falls.

**Table 1:** Characteristics of the study group.

**Characteristic**	**Category/measure**	**Value**
Age	Mean (years)	76.9
Age	Median (years)	73.0
Gender	Women (%)	76.9
Gender	Men (%)	23.1
Residence	Urban (%)	64.4
Residence	Rural (%)	35.6
Education	Primary (%)	15.4
Education	Vocational (%)	22.8
Education	Secondary/Technical (%)	49.3
Education	Higher (%)	12.6
Work Type	Intellectual (%)	54.7
Work Type	Physical (%)	45.3
Falls	Mean number	1.77
Falls	Median number	2.0

Participants aged 60 and older were included to capture early aging processes that may contribute to fall risk, particularly in individuals transitioning from middle to older adulthood. Participants were recruited from patients attending the Rehabilitation Clinic and Center as outpatients during January 2020 and February 2021. Hospitalized patients were excluded to ensure the study focused on individuals capable of participating in community-based fall prevention strategies. All patients were informed about the study's objectives and provided written consent. The criterion of “minimal assistance in movement” referred to the ability to move using aids such as canes or walkers, while severe mental disorders were assessed using the Mini-Mental State Examination (MMSE), with scores below 24 leading to exclusion. The Mini-Mental State Examination (MMSE) used in this study was a validated Polish version. The adaptation and validation process has been described by Magierska et al. (23). The average age of the respondents was 73.01 ± 6.46 years. The study was conducted once, immediately after the patient's admission to the clinic or rehabilitation center. The research was carried out between January 2020 and February 2021.

To participate in the study, individuals had to be 60 years of age or older and have experienced a fall. Detailed inclusion and exclusion criteria are presented in Table 2. The exclusion criteria were designed to reduce confounding factors that could obscure the relationship between socioeconomic factors and fall risk. For instance, individuals with severe neuro-logical or psychiatric conditions were excluded because their fall risk is primarily driven by clinical, rather than socio-economic, factors. This approach allowed the study to focus specifically on the socioeconomic determinants of falls.

**Table 2:** Inclusion and exclusion criteria.

**Inclusion criteria**	**Exclusion criteria**
Individuals aged 60 years or older.	Severe neurological conditions (e.g., advanced Parkinson's disease, dementia).
Experienced at least one fall within the past year.	Mental disorders impeding cooperation (assessed using MMSE; scores below 24 led to exclusion).
Ability to move independently or with minimal assistance (e.g., using canes or walkers).	Hospitalization at the time of the study.
Written consent to participate in the study.	Lack of written consent to participate in the study.

All procedures were conducted personally by the study authors, ensuring consistency in data collection and measurements.

### 2.2 Research methodology

The diagnostic survey method was employed in this study. A custom-designed questionnaire was used, featuring primarily dichotomous questions offering two response options, as well as multiple-choice questions. The full questionnaire, consisting of 58 questions, is provided as Supplementary material. It included sections addressing participants' socioeconomic conditions, health status, and previous fall experiences. The questionnaire comprised 58 closed-ended questions, of which 56 were single-choice and 2 were multiple-choice. The age of the participants was determined based on their year of birth. For the purposes of the study, respondents were categorized into the following age groups: early old age (60–74 years), old age (75–89 years), and advanced old age (90 years and above). This classification is based on previous gerontological studies defining age brackets within the older adult population (24). The questionnaire included questions addressing the socioeconomic and health aspects of the patients' lives. Participants were divided into four categories to analyze the frequency and characteristics of falls: “First-ever fall,” “One fall in a year,” “Two falls in a year,” and “Three or more falls in a year.” Falls were categorized as single-event falls, recurrent falls (two falls in a year), and frequent falls (three or more falls in a year), following the criteria established by (25). The “First-ever fall” group included individuals who experienced their very first fall in their lifetime during the study year, with no prior history of falls. If a participant experienced their first two falls within the study year, they were categorized under “Two falls in a year”. This classification allowed for a nuanced analysis of how socioeconomic factors influence different patterns of fall frequency. The term “First-ever fall” was defined as the very first fall experienced by a participant in their lifetime, reported during the study. This is distinct from the term “one fall in a year,” which refers to participants who experienced exactly one fall in the year preceding the study but had a history of previous falls in earlier years. This distinction allowed for a more nuanced analysis of fall frequency and its potential relationship with socioeconomic factors. This article focuses exclusively on the socioeconomic content.

### 2.3 Statistical analysis methods

Responses obtained from the questionnaires were recorded in Microsoft Excel 2010 and subsequently analyzed using the Statistica software, version 13.5 (Tibeco). Relation-ships between categorized variables were analyzed using the chi-square test. Multivariate analysis was not conducted in the initial phases due to the exploratory nature of this study and the sample size limitations. Odds ratio calculations were considered; however, Poisson regression was chosen as the preferred method due to its suitability for count data. However, a Poisson regression analysis was per-formed to assess the impact of selected socioeconomic factors, including age groups, type of work, and financial problems, on the annual number of falls. This method was chosen due to the count-based nature of the dependent variable (number of falls). For numerical data that did not exhibit a normal distribution, the nonparametric Mann-Whitney U test was applied. Results with a *p*-value of < 0.05 were considered statistically significant. Additionally, the normality of distribution was verified using the Shapiro-Wilk test.

## 3 Results

During the analysis, the data of the study participants were divided into two groups based on gender (women and men) and three groups based on age range. In the early old age group, 64.1% of participants were included, followed by 29.6% in old age and 6.3% in advanced old age. Among the respondents, 64.4% were city dwellers, while 35.6% lived in rural areas. Married individuals constituted 62.3% of the group, widowed individuals 33.6%, singles 2.8%, and divorced or separated individuals 2.3%.

Regarding occupational background, 55.0% of the participants had previously worked in intellectual professions, while 45.0% had worked in physical labor. Nearly half of the respondents (49.3%) had secondary/post-secondary education, 22.8% had vocation-al education, 15.4% had primary education, and only 12.6% had higher education. The majority of participants (56.1%) lived in two-person households, 33.1% lived alone, and 10.8% lived in households with more than two members. For almost 95.0% of the respondents, the main source of income was a pension or retirement benefits, while only 5.0% relied on income from employment.

Among all participants, the largest group consisted of individuals who experienced their first-ever fall (38.2%). For 17.0% of the respondents, it was a single fall during the year of the study, 32.2% experienced two falls that year, and 12.5% experienced three or more falls. Men were more likely than women to have experienced their first-ever fall (43.2% vs. 36.6%) and a single fall during the study year (18.5% vs. 16.6%). Conversely, a higher percentage of women than men experienced a second fall in the same year (32.6% vs. 30.8%) and especially a third or subsequent fall (14.6% vs. 7.4%). However, these differences were not statistically significant (*p* = 0.61).

In the early old age group, 50.2% experienced their first-ever fall, 15.1% experienced a single fall in the year, 26.2% experienced two falls in the year, and 8.4% experienced three or more falls during the year. Among those in old age, 17.3% experienced their first-ever fall, 22.2% experienced a single fall in the year, 42.3% experienced two falls, and 18.3% experienced three or more falls in a year. In advanced old age, 13.6% experienced their first-ever fall and a single fall in the year, 45.5% experienced two falls, and 27.3% experienced three or more falls within 12 months. These differences were found to be statistically significant (*p* = 0.00001) (Table 3).

**Table 3:** Frequency of falls considering the age of the studied individuals.

**Frequency of falls**		**Early old age**	**Old age**	**Advanced old age**	**Total**
First-ever fall	*n*	113	18	3	134
	%	50.22	17.31	13.64	38.18
One fall in a year	*n*	34	23	3	60
	%	15.11	22.12	13.64	17.09
Two falls in a year	*n*	59	44	10	113
	%	26.22	42.31	45.45	32.19
Three or more falls in a year	*n*	19	19	6	44
	%	8.44	18.27	27.27	12.54
Total	*n*	225	104	22	351
	%	64.10	29.63	6.27	100.00
Chi-square test (*p*-value)	0.00001				

More urban residents experienced their first-ever fall compared to rural residents (41.2% vs. 32.8%). More rural residents compared to urban residents experienced one fall, two falls, and three or more falls (18.4% vs. 16.4%, 35.2% vs. 30.5%, and 13.6% vs. 11.9%, respectively) higher percentage. These differences were not statistically significant (*p* = 0.49). The highest percentage of first-ever falls occurred among married individuals (48.4%), followed by widowed individuals (22.8%), single individuals (20.0%), and divorced or separated individuals (12.5%). One fall in the preceding year was most common among divorced or separated individuals (50.0%), followed by single individuals (20.0%), widowed individuals (16.9%), and married individuals (15.8%). Two falls were most frequent among single individuals (40.0%), followed by widowed individuals (38.2%) and divorced/separated individuals (38.2%), with married individuals being the least affected (28.4%). Three or more falls were most common among widowed individuals (23.0%) and single individuals (20.0%), and least common among married individuals (7.4%). These differences were statistically significant (*p* = 0.00002). It was found that single individuals were the most commonly affected by falls. The highest percentage of first-ever falls occurred among individuals with higher education (54.5%), followed by those with secondary/post-secondary education (42.7%) and vocational education (33.7%), while the lowest percentage was among individuals with primary education (16.7%).

Similarly, one fall in the preceding year was most common among individuals with higher education (27.2%), with slightly lower rates among those with secondary/post-secondary education (16.2%), vocational education (15.0%), and primary education (14.8%).

Two falls in the study year were most frequent among individuals with primary education (46.3%), followed by those with vocational education (40.0%) and secondary/post-secondary education (29.0%), and least frequent among individuals with higher education (11.4%). Three or more falls were most common among individuals with primary education (22.2%), followed by those with secondary/post-secondary education (11.5%) and vocational education (11.3%), and least common among individuals with higher education (6.8%). These differences were statistically significant (*p* = 0.00015). The highest percentage of first-ever falls occurred among individuals engaged in intellectual work compared to those in physical labor (46.8% vs. 27.7%). Similarly, one fall in the preceding year was more common among intellectual workers than physical laborers (18.2% vs. 15.7%). Conversely, both two falls in a year (41.5% vs. 24.5%) and three or more falls (15.1% vs. 10.4%) were more frequent among physical laborers than intellectual workers. These differences were statistically significant (*p* = 0.00039) (Table 4).

**Table 4:** Frequency of falls by type of work performed by participants.

**Frequency of falls**		**Intellectual work**	**Physical work**	**Total**
First-ever fall	*n*	90	44	134
	%	46.88	27.67	38.18
One fall in a year	*n*	35	25	60
	%	18.23	15.72	17.09
Two falls in a year	*n*	47	66	113
	%	24.48	41.51	32.19
Three or more falls in a year	*n*	20	24	44
	%	10.42	15.09	12.54
Total	*n*	192	159	351
	%	54.70	45.30	100.00
Chi-square test (*p*-value)	0.0004			

The first-ever fall occurred most commonly among individuals living in two-person households (47.2%) and those living in larger households (34.2%), and least frequently among individuals living alone (24.1%). One fall in the year preceding the study most often occurred among individuals living alone (19.8%), followed by those in two-person households (16.2%) and least frequently among those in larger households (16.2%). Two falls in the year affected the largest percentage of individuals living alone (40.5%), followed by those in larger households (31.6%), and the smallest percentage of individuals in two-person households (27.4%). Three or more falls in a year most commonly affected individuals in larger households (21.1%), followed by those living alone (15.5%), and least frequently those in two-person households (9.1%). These differences were statistically significant (*p* = 0.002).

Among respondents whose primary source of income was a pension or retirement benefits, 36.1% experienced their first-ever fall, 16.8% experienced one fall in the year, 34.0% experienced two falls, and 12.9% experienced three or more falls in a year. Among respondents whose source of income was their own employment, 78.6% experienced their first-ever fall, and 21.4% experienced one fall in the year. Among respondents whose income was derived from their spouse's earnings, all experienced their first-ever fall (100.0%). Among those supported by social assistance, 100.0% experienced three or more falls in a year. Among respondents whose primary income came from other sources, 50.0% experienced their first-ever fall, and 50.0% experienced one fall in the year. These differences were statistically significant (*p* = 0.004).

Among individuals who experienced their first-ever fall, the mean score on the financial problems index resulting in unmet health needs was 1.04±1.72 (median = 0; range: 0–8 points). For individuals who experienced one fall in the year, the mean score on this index was 1.77 ± 2.08 (median = 1.5; range: 0–8 points). Among those who experienced two falls in the year, the mean score was 2.09±1.76 (median = 2; range: 0–8 points). Among those who experienced three or more falls, the mean score was the highest at 3.2 ± 2.58 (median = 3; range: 0–8 points). These differences were statistically significant (*p* = 0.00001) (Table 5).

**Table 5:** Financial constraints and their association with fall frequency.

**Frequency of Falls**	***M***	***N***	**SD**	**Min**.	**Max**.	**25th Quartile**	**Me**	**75th Quartile**
First-ever fall	1.04	134	1.72	0	8	0	0	2
One fall in a year	1.77	60	2.08	0	8	0	1.5	2
Two falls in a year	2.09	113	1.76	0	8	1	2	2
Three or more falls in a year	3.20	44	2.58	0	8	1.5	3	4
Overall	1.77	351	2.04	0	8	0	2	2
Kruskal-Wallis *p*-value	0.00001							
ANOVA *p-*value	0.000000001							
**Pairwise comparisons (mean differences)**								
	**{1}—*****M** =* **1.0373**	**{2}—*****M** =* **1.7667**	**{3}—*****M** =* **2.0885**	**{4}—*****M** =* **3.2045**
First-ever fall {1}		0.015065	0.000024	0
One fall in a year{2}	0.015065		0.29525	0.000192
Two falls in a year{3}	0.000024	0.29525		0.001192
Three or more falls {4}	0	0.000192	0.001192	

The Table 6 presents the results of the Poisson regression analysis assessing the impact of age groups, type of work, and financial problems on the number of falls per year.

**Table 6:** Results of the Poisson regression analysis assessing the impact of age groups, type of work, and financial problems on the number of falls per year.

**Variable**	**Coefficient (Coef.)**	**Standard error (Std.Err.)**	***Z*-Value (*Z*)**	***p*-Value (*P*>|*z*|)**	**95% confidence interval (lower, upper)**
Intercept	0.1818	0.094	1.925	0.054	(−0.003, 0.367)
Old age	0.0865	0.092	0.938	0.348	(−0.094, 0.267)
Advanced old age	0.3304	0.176	1.877	0.061	(−0.015, 0.676)
Physical work	0.0777	0.088	0.888	0.375	(−0.094, 0.249)
Financial problems	0.3325	0.092	3.625	0.000	(0.153, 0.512)

The conducted Poisson regression analysis assessed the impact of age groups, type of work performed, and financial problems on the annual number of falls. The intercept represents the predicted number of falls for individuals in the reference group, namely those in the Early Old Age, performing intellectual work, and not reporting financial problems.

Compared to the reference group (Early Old Age), individuals in the Old Age and Advanced Old Age groups did not differ significantly in terms of the number of falls (*p* > 0.05). Type of work: Differences between individuals performing physical work and intellectual work were not statistically significant (*p* = 0.375), indicating a similar number of falls in both groups. Financial problems: Individuals reporting financial problems had, on average, a 33.25% higher number of falls than those without such issues (*p* < 0.001).

## 4 Discussion

The frequency of falls among older adults increases with age (26, 27). After the age of 65, ~35.0–40.0% of independently functioning seniors experience a fall, while among those aged 80 and older, falls affect nearly 50.0%. Women are three times more likely to experience a fall than men (8, 28). Analysis of medical records from the Emergency Department of Independent Public Clinical Hospital No. 1 (SPSK 1) in Lublin confirmed an increase in fall frequency with age: 29.7% of individuals aged 65–74 years experienced a fall, compared to 40.6% of those aged 75–89 years (29, 30). Other Polish studies conducted among 4,920 individuals aged 65 and older also demonstrated a proportional increase in fall frequency with age. These studies further confirmed that falls occurred higher percentage among women than men (22.7% vs. 13.2%) (31). Our study found that urban residents were more likely to experience their first-ever fall, whereas rural residents had a higher likelihood of experiencing multiple falls. These findings align with prior research, which suggests that differences in infrastructure, accessibility to medical care, and lifestyle factors may contribute to variations in fall risk between urban and rural populations.

In the present study, among those who experienced falls, nearly 65.0% were aged 60–74 years, 30.0% were aged 75–89 years, and just over 6.0% were aged 89 years and older. This distribution reflects the specific criteria used for selecting the study group. The observed gender differences in fall risk, with women experiencing falls more frequently than men, are consistent with previous studies. These differences may be attributed to factors such as higher life expectancy among women, greater prevalence of osteoporosis, and gender-related behavioral differences in reporting falls.

The findings confirm that the frequency of falls increases with age. While many risk factors identified in this study align with previous research, its novelty lies in the focus on the socioeconomic determinants of fall frequency in the unique context of Poland. Our findings highlight the strong association between fall risk and socioeconomic factors, including education level, prior occupational activity, and financial status. Older adults with lower education levels and those engaged in physical labor were at a significantly higher risk of falls. Financial instability was another key determinant, suggesting that economic constraints may limit access to fall prevention measures. These findings emphasize the need for targeted interventions that address both educational and financial barriers to fall prevention. The study highlights disparities in fall risk associated with education, prior occupational activity, and financial status, which are shaped by localized socioeconomic and cultural conditions. The observed disparities in fall risk may be linked to the structure of the Polish healthcare system, where access to preventive geriatric care is often limited, particularly for individuals with lower financial means. Additionally, Poland's socioeconomic landscape, characterized by significant income disparities and regional differences in access to rehabilitation services, may further influence fall incidence. These insights provide a basis for targeted public health interventions in Poland. In Poland, older adults with lower educational attainment and prior engagement in physically demanding occupations may have limited awareness of fall prevention strategies. Financial instability further exacerbates fall risk, as many older people cannot afford necessary home modifications or assistive devices. Additionally, cultural attitudes toward aging and rehabilitation influence the likelihood of seeking medical assistance after a fall. Three or more falls were experienced by 8.5% of individuals in early old age, com-pared to more than three times as many (27.0%) among those in advanced old age. The results also confirmed that women experience falls more often than men. Three or more falls in a year were reported by 8.0% of men and nearly twice as many women (over 14.0%). Similar results were obtained in a Spanish study, which showed that women were 2.5 times more likely to experience falls than men (32). In another study on the frequency of falls and their risk factors conducted among 103 individuals aged 65 years, women were more likely to experience falls than men (50.0% vs. 28.0%) (33). Similarly, another Polish study among 105 individuals aged 75–89 years also found that women experienced falls higher percentage than men (65.4% vs. 44.0%) (24). The PolSenior study also con-firmed that women are more likely to experience falls and that the frequency of falls in-creases with age (34).

Chinese studies conducted among individuals aged over 60 years, both urban and rural, showed that 13.0% of urban residents and over 11.0% of rural residents experienced falls. The frequency of falls was higher among women than men, in both urban and rural areas (35). Similarly, a study conducted in Tehran among individuals over 65 years old found that ~40.0% of this population experienced falls, with women affected higher percentage than men (36). Studies conducted in Saudi Arabia also found that women experienced falls higher percentage than men (34.5% vs. 28.5%) (37).

Contrary results were obtained from an analysis of medical records at the Provincial Integrated Hospital in Szczecin between 2006 and 2017. During this period, 2,330 individuals were treated for accidents, 96.2% of which were falls. Men experienced falls more often than women (53.4% vs. 46.6%) and individuals over 65 years old accounted for 61.6% of the cases (38). Similarly, studies on the risk of falls among hospitalized individuals in Brazil showed that men experienced falls more often than women, but this was attributed to the predominance of men in the study group (39).

Research conducted in Wrocław among older women from different residential environments found that rural women had a more than four-fold higher fall risk index (*Z*-score = 2.7) compared to urban women (*Z*-score = 0.6) (8, 40).

Dutch studies among individuals aged 65 years and older found that ~30.0% experienced at least one fall annually, while 15.0% experienced two or more falls (41, 42).

The present study among patients at the Institute of Rural Health in Lublin showed that in the year preceding the study, ~38.0% experienced their first-ever fall, 17.0% experienced one fall, just over 32.0% experienced two falls, and ~13.0% experienced three or more falls. (30).

Studies analyzing the causes and consequences of falls among women aged over 50 years living alone in Kraków showed that ~30.0% experienced at least one fall in the past year. In a randomly selected group of 100 women with an average age of 63.9 years, 62.0% experienced one fall, 25.0% two falls, 8.0% three falls, and 5.0% four or more falls in the year preceding the study (43).

Research conducted among older rural residents showed that 21.0% experienced no falls, 28.0% experienced one fall annually, and 51.0% experienced more than one fall annually (44). Another study on the causes, frequency, and injuries associated with falls among 100 geriatric patients found similar results: 73.0% experienced 1–2 falls, 7.0% experienced 3–4 falls, 10.0% experienced 5–6 falls, 2.0% experienced 7–8 falls, and 8.0% re-ported 10 or more falls (45).

The present study showed that first-ever falls were more common among urban residents than rural residents (41.0% vs. 33.0%), while three or more falls were slightly more frequent among rural residents than urban residents (13.6% vs. 12.0%). The significance of this study lies in its focus on the relationship between socioeconomic factors and the frequency of falls among older adults in Poland, a country with unique healthcare and social structures. While the findings align with previous international studies, they highlight critical disparities in fall risk related to education level, prior occupational activity, and financial status, which may be influenced by localized socioeconomic and cultural con-texts. These results underline the importance of tailored prevention strategies that address the specific needs of older adults in Poland. The most significant factor turned out to be financial problems, which had a substantial impact on the frequency of falls. This finding highlights the necessity of providing economic support to groups with lower financial status to reduce the risk of falls. Unlike many existing studies, this research emphasizes the socioeconomic determinants of fall frequency, offering a basis for public health interventions that prioritize education and financial support for vulnerable populations.

### 4.1 Study limitations

This study has certain limitations. Data were collected at a single point in time, making it impossible to track long-term changes in fall frequency. The study relied on participants' self-reports, which may involve memory biases or underreporting of falls. Another limitation is the lack of full control over external variables that could influence the results, such as participants' health status, physical activity levels, or access to healthcare. Furthermore, the study was conducted in a single center in Poland, which may limit the generalizability of the findings to other populations.

## 5 Conclusions

Falls among older adults are influenced by multiple factors, including age, gender, education level, and socioeconomic status. Age significantly increases fall frequency, and women are more prone to falls than men, consistent with global findings. Additionally, environmental and socioeconomic factors, such as financial constraints and prior occupational activity, play a crucial role in fall risk.

### 5.1 Practical implications

The findings of this study highlight the importance of addressing socioeconomic disparities in fall prevention strategies. Further research should include multivariate analyses and consider additional factors such as physical activity levels, access to healthcare, and environmental barriers. These insights will contribute to the development of targeted interventions to mitigate fall risks among older adults in Poland. Future studies should incorporate multivariate analyses to assess additional factors such as physical activity levels, access to healthcare, and environmental barriers, to develop more targeted fall prevention strategies.

## Data Availability

The original contributions presented in the study are included in the article/Supplementary material, further inquiries can be directed to the corresponding author.
