# Supplementary material for: Risk factors and socioeconomic determinants of falls among older adults
Source: Front Public Health. 2025 Mar 13;13:1571312. doi: 10.3389/fpubh.2025.1571312 (PMC11966436; doi:10.3389/fpubh.2025.1571312)
Supplement: Supplementary file 1 [file Table_1.docx]

**Appendix**

**Author's Questionnaire**
Survey number: □
Name and surname: ________________________________________

1. Year of birth: □□□□
2. Gender: (1 - female, 2 - male) □
3. Place of residence: (1 - city, 2 - village) □
4. Marital status:
   (1 - single, 2 - married, 3 - widowed, 4 - divorced/separated, 5 - informal relationship) □
5. Education:
   (1 - primary, 2 - vocational, 3 - secondary/post-secondary, 4 - higher education/bachelor's degree) □
6. Current occupation: (1 - white-collar worker, 2 - manual laborer) □
7. Number of people in the household (including the participant): □
8. Main source of income:
   (1 - pension/retirement, 2 - personal employment income, 3 - spouse’s income, 4 - welfare, 5 - family support, 6 - other sources of income) □
9. BMI = body weight: ______ kg / height: ______ m²
10. Do you have any neurological diseases? (1 - yes, 2 - no) □
11. Do you have any cardiovascular diseases? (1 - yes, 2 - no) □
12. Do you have any vestibular disorders? (1 - yes, 2 - no) □
13. Do you have any musculoskeletal disorders (e.g., osteoarthritis, osteoporosis)? (1 - yes, 2 - no) □
14. Do you have any metabolic diseases (e.g., diabetes, thyroid diseases)? (1 - yes, 2 - no) □
15. Do you have any eye diseases (e.g., cataract, glaucoma, macular degeneration)? (1 - yes, 2 - no) □
16. Do you take medications to lower blood pressure regularly? (1 - yes, 2 - no) □
17. Do you take medications to lower blood sugar levels regularly? (1 - yes, 2 - no) □
18. Do you take diuretics regularly? (1 - yes, 2 - no) □
19. Do you take sedative/psychotropic medications? (1 - yes, 2 - no) □
20. How many medications do you take daily? □□
21. Do you take only medications prescribed by a doctor? (1 - yes, 2 - no) □
22. Are you satisfied with your health status?
    (1 - very satisfied, 2 - satisfied, 3 - moderately satisfied, 4 - moderately dissatisfied, 5 - dissatisfied, 6 - very dissatisfied) □
23. Are you satisfied with safety in your place of residence?
    (1 - very satisfied, 2 - satisfied, 3 - moderately satisfied, 4 - moderately dissatisfied, 5 - dissatisfied, 6 - very dissatisfied) □
24. Are you satisfied with your economic status?
    (1 - very satisfied, 2 - satisfied, 3 - moderately satisfied, 4 - moderately dissatisfied, 5 - dissatisfied, 6 - very dissatisfied) □
25. In the past year, has your household lacked funds for:
    a) fulfilling prescriptions? (1 - yes, 0 - no) □
    b) dental treatments? (1 - yes, 0 - no) □
    c) obtaining dental prosthetics? (1 - yes, 0 - no) □
    d) doctor visits? (1 - yes, 0 - no) □
    e) medical/laboratory tests? (1 - yes, 0 - no) □
    f) rehabilitation treatments? (1 - yes, 0 - no) □
    g) spa treatments? (1 - yes, 0 - no) □
    h) hospital treatments? (1 - yes, 0 - no) □
    Final score: □ /8 points
26. To what extent are you socially engaged?
    a) participation in a university of the third age: (1 - yes, 0 - no) □
    b) professional activity: (1 - yes, 0 - no) □
    c) unpaid work (volunteering): (1 - yes, 0 - no) □
    d) activities for the local community: (1 - yes, 0 - no) □
    e) physical activity/sports: (1 - yes, 0 - no) □
    f) computer use: (1 - yes, 0 - no) □
    g) internet use: (1 - yes, 0 - no) □
    h) participation in social gatherings: (1 - yes, 0 - no) □
    Final score: □ /8 points
27. What type of sport/physical exercises do you practice?
    a) aerobics/gymnastics □
    b) Nordic walking/walking □
    c) cycling □
    d) other activities (e.g., gardening) □
28. How do you rate access to medical services?
    (1 - very good, 2 - good, 3 - average, 4 - poor, 5 - very poor) □
29. How do you rate access to social services?
    (1 - very good, 2 - good, 3 - average, 4 - poor, 5 - very poor) □
30. How many times have you fallen in the past year? □
31. Was this your first fall? (1 - yes, 2 - no) □
32. Did your last fall occur: (1 - at home, 2 - outside the home) □
33. If the fall occurred at home, where did it happen?
    (1 - kitchen, 2 - bedroom, 3 - bathroom, 4 - stairs, 5 - other room, 6 - not applicable) □
34. If the fall occurred outside the home, where did it happen?
    (1 - street/sidewalk, 2 - farm area, 3 - hospital/care home, 4 - service premises, e.g., store, hairdresser, 5 - not applicable) □
35. What time did your last fall occur?
    (1 - 6:00–10:00, 2 - 10:00–14:00, 3 - 14:00–18:00, 4 - 18:00–22:00, 5 - 22:00–6:00) □
36. What activity were you performing during your last fall?
    (1 - standing up, 2 - sitting down, 3 - walking, 4 - bending down, 5 - turning, 6 - moving on stairs, 7 - slipping from a standing position, 8 - bathing, 9 - carrying objects) □
37. What were the direct causes of your last fall?
    (1 - fainting or loss of consciousness, 2 - drop in blood pressure, 3 - heart rhythm disturbances, 4 - vision disorders, 5 - seizures, 6 - dizziness, 7 - balance disorders, 8 - gait disorders, 9 - general frailty, 10 - drop in blood glucose levels, 11 - shortness of breath/chest pain, 12 - other internal causes) □□
38. Have you suffered a head injury as a result of any fall? (1 - yes, 2 - no) □
39. Have you suffered a bone fracture as a result of any fall? (1 - yes, 2 - no) □
40. What part of your body was injured during the last fall?
    (1 - head, 2 - torso, 3 - left upper limb, 4 - right upper limb, 5 - left lower limb, 6 - right lower limb) □
41. Was hospitalization required as a result of any fall? (0 - no, 1 - yes) □
42. Was medical assistance provided only at the scene of the fall? (0 - no, 1 - yes) □
43. Was the assistance of others necessary due to any fall? (0 - no, 1 - yes) □
44. Was inappropriate footwear or clothing a cause of your fall(s)? (1 - yes, 2 - no) □
45. Were unsuitable aids or assistive devices for walking a cause of your fall(s)? (1 - yes, 2 - no) □
46. Was improper or insufficient lighting a cause of your fall(s)? (1 - yes, 2 - no) □
47. Was a slippery or wet floor a cause of your fall(s)? (1 - yes, 2 - no) □
48. Was tripping over thresholds, objects, or carpets a cause of your fall(s)? (1 - yes, 2 - no) □
49. Do you use non-slip mats? (1 - yes, 2 - no) □
50. Do you use handrails in the bathroom? (1 - yes, 2 - no) □
51. Were adverse weather conditions a cause of your fall(s)? (1 - yes, 2 - no) □
52. Do you encounter architectural barriers in your environment (e.g., high thresholds, curbs, stairs without railings)? (1 - yes, 2 - no) □
53. Has your level of fear increased, and has your physical fitness deteriorated after experiencing falls? (1 - yes, 2 - no) □
54. Have you started limiting your physical activity after experiencing falls? (1 - yes, 2 - no) □
55. Do you use the help of a caregiver? (1 - yes, 2 - no) □
56. How does your health condition after the fall(s) affect your relationships with others?
    (1 - positively, 2 - negatively, 3 - no impact) □
57. How does your health condition after the fall(s) affect your daily life?
    (1 - positively, 2 - negatively, 3 - no impact) □
58. How does your health condition after the fall(s) affect handling formal matters (e.g., at offices)?
    (1 - positively, 2 - negatively, 3 - no impact) □
